# Volatile Anesthetics Influence Blood-Brain Barrier Integrity by Modulation of Tight Junction Protein Expression in Traumatic Brain Injury

**DOI:** 10.1371/journal.pone.0050752

**Published:** 2012-12-10

**Authors:** Serge C. Thal, Clara Luh, Eva-Verena Schaible, Ralph Timaru-Kast, Jana Hedrich, Heiko J. Luhmann, Kristin Engelhard, Christoph M. Zehendner

**Affiliations:** 1 Department of Anesthesiology, University Medical Center of the Johannes Gutenberg-University, Mainz, Germany; 2 Institute of Physiology and Pathophysiology, University Medical Center of the Johannes Gutenberg-University, Mainz, Germany; Biological Research Centre of the Hungarian Academy of Sciences, Hungary

## Abstract

Disruption of the blood-brain barrier (BBB) results in cerebral edema formation, which is a major cause for high mortality after traumatic brain injury (TBI). As anesthetic care is mandatory in patients suffering from severe TBI it may be important to elucidate the effect of different anesthetics on cerebral edema formation. Tight junction proteins (TJ) such as zonula occludens-1 (ZO-1) and claudin-5 (cl5) play a central role for BBB stability. First, the influence of the volatile anesthetics sevoflurane and isoflurane on in-vitro BBB integrity was investigated by quantification of the electrical resistance (TEER) in murine brain endothelial monolayers and neurovascular co-cultures of the BBB. Secondly brain edema and TJ expression of ZO-1 and cl5 were measured in-vivo after exposure towards volatile anesthetics in native mice and after controlled cortical impact (CCI). In in-vitro endothelial monocultures, both anesthetics significantly reduced TEER within 24 hours after exposure. In BBB co-cultures mimicking the neurovascular unit (NVU) volatile anesthetics had no impact on TEER. In healthy mice, anesthesia did not influence brain water content and TJ expression, while 24 hours after CCI brain water content increased significantly stronger with isoflurane compared to sevoflurane. In line with the brain edema data, ZO-1 expression was significantly higher in sevoflurane compared to isoflurane exposed CCI animals. Immunohistochemical analyses revealed disruption of ZO-1 at the cerebrovascular level, while cl5 was less affected in the pericontusional area. The study demonstrates that anesthetics influence brain edema formation after experimental TBI. This effect may be attributed to modulation of BBB permeability by differential TJ protein expression. Therefore, selection of anesthetics may influence the barrier function and introduce a strong bias in experimental research on pathophysiology of BBB dysfunction. Future research is required to investigate adverse or beneficial effects of volatile anesthetics on patients at risk for cerebral edema.

## Introduction

The blood-brain barrier (BBB) is a highly organized endothelial barrier, which separates the central nervous system (CNS) from peripheral circulation [1]. Disruption of the BBB and consecutive vasogenic brain edema formation is one of the most deleterious sequelae of traumatic brain injury (TBI) [2]. As a result, intracranial pressure (ICP) may increase and contribute to the development of secondary brain damage [3]. Pronounced edema formation might finally lead to therapy resistant ICP increase causing suspension of cerebral perfusion and finally leading to brain death. Tight junction proteins (TJ) are a hallmark for the integrity of the BBB that essentially contribute to its structural inviolacy. Thereby paracellular flux of hydrophilic molecules across the BBB is limited, which is essential for the functional integrity of the CNS [4]. The TJ form a complex of transmembrane (junctional adhesion molecule-1, occludin, and claudins) and cytoplasmic (zonula occludens-1 [ZO-1], zonula occludens-2 [ZO-2], cingulin, AF-6, and 7H6) proteins, which are linked to the actin cytoskeleton [5]. The development of vasogenic brain edema after TBI is caused by opening of the BBB and consists of protein-rich fluid [5]. The alteration of TJ assembly may contribute to the loss of BBB integrity and BBB breakdown [6]. It has been suggested that different anesthetics are capable of modulating BBB permeability [7]–[9]. As anesthetic care in patients suffering from severe traumatic brain injury is mandatory it may be important to elucidate the effect of different anesthetics on cerebral edema formation after TBI.

In the present study, the effect of the volatile anesthetics isoflurane and sevoflurane on barrier integrity was investigated in an in-vitro BBB model, in-vivo in native animals and following experimental TBI to determine the impact of anesthetic exposure on BBB integrity, development of vasogenic brain edema and TJ protein integrity and expression after TBI.

## Methods

### Cell Culture

The murine brain endothelial cell (BEC) line bEnd.3 (American Type Culture Collection, Manassas, VA, USA) was used as a BBB model and cultured as recommended by the manufacturer. Cell cultures were maintained at a humidified atmosphere, 37°C and 5% CO_2_. Media consisted of DMEM Glutamax supplemented with 2% Penicillin/Streptomycin (Invitrogen GmbH, Karlsruhe, Germany) and 15% fetal calf serum (Biochrom AG, Berlin, Germany). bEnd.3 passages 10–19 were used for experimental manipulations.

### Neurovascular co-culture model consisting of cortical organotypic slice cultures (COSC) and brain endothelial cells

Co-culture procedures were carried out as described in detail before with slight modifications regarding COSC media [10], [11]. All experiments were carried out according to the national and European (86/609/EEC) laws for use of animals in research and approved by the institutional committee for animal care. Great care was taken to minimize the amount of animals and their suffering. In brief, COSC from C57/Bl6 postnatal day 3 or 4 mice were prepared according to the Stoppini method [12]. COSC medium was composed of 50% MEM HEPES Glutamax containing 1 mg/ml glucose (Invitrogen GmbH, Karlsruhe, Germany), 25% heat inactivated horse serum, 25% HBSS supplemented with calciumchloride 2 mmol/l and magnesiumchloride 1 mmol/l. Glucose was added to reach a concentration of about 7 mg/ml. No antibiotics were used. The pH of the medium was carefully adjusted to 7.2 at 37°C. COSC were kept in culture for 3 days before being used for the co-culture. Slices were carefully removed from their membranes as described in detail before [10]. bEnd.3 media was used for co-culture procedures in ECIS arrays. COSC were thoroughly examined for integrity with light microscopy before using them for co-culture procedures.

### Anesthetic in-vitro delivery system

The 8-well plates containing BEC or bEnd.3 and COSC co-cultures were placed in an exposure chamber on a heating pad and heated to 37°C. The concentrations of volatile anesthetics adjusted with a calibrated isoflurane or sevoflurane vaporizer (Draeger, Germany) and were confirmed by sampling the solution of the wells and assessing the anesthetic concentration in the solution by headspace technique using a gas chromatograph (Hewlett Packard 5890 Series II, USA).

### Electrical Cell Substrate Impedance Sensing

BEC were seeded at a density of 50,000–80,000 cells per well in an 8-well gold electrode assay (0.8 cm^2^ growth area, from ibidi in cooperation with Applied BioPhysics, Martinsried, Germany). For co-culture procedures COSC were combined with bEnd.3 similarly as described before [10]. When trans endothelial electrical resistance (TEER) measurements reached maximal values cells were exposed to 1.5% of isoflurane, 3.5% of sevoflurane or room air (air_R_) for 20 min as described in the previous paragraph. A calibrated vaporizer was used for this purpose (Draeger, Germany). Thereafter bEnd.3 and co-cultures were maintained at 37°C and 5% CO_2_ for 24 hours in a humidified atmosphere. On average bEnd.3 monolayers reached TEER values of 841.71±129.94 Ohm (0.8 cm^2^ growth area). TEER in co-cultures consisting of COSC and bEnd.3 monolayers was higher than in monocultures. Here, TEER was 1171.83±81.21 Ohm (0.8 cm^2^ growth area) on average. Only probes that reached at least 700 Ohm were used for experiments.

24 hours after gas exposure TEER values were evaluated again and set in relation to respective TEER values before gas exposure.

### Experimental Animals

Male C57Bl6N mice (weight: 18 to 24 g, Charles River Laboratory, Sulzfeld, Germany) were investigated. Before and during experiments animals were cared for in compliance with institutional guidelines of the Johannes Gutenberg-University, Mainz. The Animal Ethics Committee of the Landesuntersuchungsamt Rheinland-Pfalz approved all experiments (protocol number: 23 177-07/G08-1-012).

### Anesthetic in-vivo regimens

Animals were randomized to three different anesthesia regimens: isoflurane, sevoflurane and no anesthesia. Anesthesia was induced in a bell jar filled with 4% isoflurane or 6% sevoflurane and maintained via facemask in spontaneously breathing animals (1.5% isoflurane or 3.5% sevoflurane in 40% O_2_ and 60% N_2_). In animals randomized to no anesthesia group an air mixture (40% O_2_ and 60% N_2_) was supplied via facemask. Native animals (no surgery) received a 20 minutes anesthesia exposure. In animals, which were subjected to brain trauma, anesthesia was maintained during the surgical preparation for about 15–20 minutes. Immediately after controlled cortical impact (CCI) or sham surgery and closure of the wound anesthesia was discontinued.

### Trauma induction

Animals were placed in a stereotactic frame and were subjected to brain trauma by CCI as previously described [13]. Briefly, after craniotomy a pneumatic contusion was induced over the right parietal cortex with a custom pneumatic controlled cortical impactor (L. Kopacz, Mainz, Germany) with following parameters: diameter 3 mm, impact velocity 8 m/s, impact duration 150 ms and the brain penetration 1 mm. Immediately after impact the craniotomy was closed and wounds were closed with filament sutures. Rectal temperature was maintained at 37°C by a feedback-controlled heating pad. After discontinuation of anesthesia mice were returned in their individual cages and allowed to recover for 4 hours in an incubator heated to 33°C and at a humidity of 35% (IC8000, Draeger, Germany). Sham-operated mice were subjected to all aspects of the protocol with exception of the trauma.

### Experimental studies

- In-vitro: Effect of isoflurane and sevoflurane on TEER: n = 8 TEER analyses for bEnd.3 monoculture procedures, n = 8 TEER analyses for neurovascular co-cultures.
- In-vivo: Effect of isoflurane and sevoflurane on brain water content and TJ protein expression in healthy animals.
  Animals (native, no surgery) were randomly assigned to three groups: (1) 1.5% isoflurane, (2) 3.5% sevoflurane, and (3) no anesthesia native animals (n = 8 per group). The anesthesia was given for 20 minutes. 24 hours after anesthesia brain water content and differential gene regulation was measured.
- In-vivo: Effect of different anesthesia protocols on brain water content after CCI.
  Hemispheric brain water content was measured 24 hours after CCI in animals randomized to: (1) isoflurane+sham surgery, (2) isoflurane+CCI, (3) sevoflurane+sham surgery or (4) sevoflurane+CCI (n = 7 per group). The anesthesia was initiated prior surgical preparation and terminated immediately after CCI and closure of the wounds.
- In-vivo: Effect of different anesthesia protocols on immunohistochemical claudin 5 (cl5) and ZO-1 protein integrity in cerebral microvessels.
  Brain trauma was induced and animals were randomly assigned to four groups: (1) isoflurane+sham surgery (n = 3), (2) sevoflurane+sham surgery (n = 3), (3) isoflurane+CCI (n = 3), (4) sevoflurane+CCI (n = 3).
- In-vivo: Effect of different anesthesia protocols on differential TJ gene expression after CCI.
  Brain trauma was induced and animals were randomly assigned to four groups: (1) isoflurane+15 min survival (n = 6), (2) sevoflurane+15 min survival (n = 6), (3) isoflurane+24 h survival (n = 8), (4) sevoflurane+24 h survival (n = 8), expression data were normalized with data from native, non-operated, and anesthetized animals (n = 6, 3 animals per anesthetic regimen).

### Brain water content

24 hours after surgery or anesthesia exposure animals were re-anesthetized with the same anesthetic regimen used during experiments and killed by decapitation. Brains were carefully removed and cut alongside the sagittal plane. Injured or unharmed right hemispheres were weighed to assess their wet weight [14]. The hemispheres were dried for 24 hours at 110°C to determine the dry weight. Based on gravimetrical differences water content was obtained by the following calculation: Hemispheric brain water content (%): (wet weight WW – dry weight DW)/WW×100.

### Tissue preparation for qPCR

For the determination of mRNA regulation, brains were gently removed and placed in a cooled (6°C) brain matrix. A 2 mm thick coronal slice was cut and separated in quadrants. The slice was defined according to the Mouse Brain Library atlas as bregma −1 mm to bregma −3 mm (www.mbl.org). The right-upper region was collected, frozen in liquid nitrogen and stored at −80°C until further processing. RNA extraction, cDNA synthesis and real-time PCR was performed with standard protocols as previously described in detail [15]. RNA concentration was spectrometrically calculated using NanoVue (GE Healthcare Europe, Munich, Germany). 0.5 µg extracted RNA was reverse-transcribed into cDNA by Verso™ cDNA Kit (ABgene, Hamburg, Germany) according to the manufacturer's instructions.

cDNA of each sample was amplified by a real-time Lightcycler 480 PCR System (Roche). Equal amounts of cDNA (1 µl) were used in duplicates and amplified with Lightcycler® 480 Probes Master or Lightcycler® 480 SYBR Green I Master (Roche). Real-time cycling parameters were as follows: thermal activation for 10 min at 95°C and 50 cycles of PCR (melting for 10 s at 95°C, annealing for 10 s at 55°C (for HybProbe assays) or according to **Table 1**, extension for 15 s at 72°C). Applied primers and probes are listed in **Table 1**. A standard curve for absolute quantification was generated with PCR DNA for each PCR product (10^1^–10^7^). The absolute copy numbers of the target genes was normalized against the absolute copy numbers of Cyclophilin A (PPIA) as control gene.

**Table 1:** Specific primer and probes and optimized temperature conditions for real-time PCR.

PCR assay (amplicon size, annealing temp)	Oligonucleotide Sequence (5′-3′)	GenBank No.
Claudin-5 (cl5)	Forw: 5′-CGTTGGAAATTCTGGGTCTG-3′	NM_013805.4
*(194 bp, 58°C, A:10s, E:15s)*	Rev: 5′-AGATTCATACACCTTGCACTG-3′	
ZO-1 isoform 1 (ZO-1 V1)	Forw: 5′-TgTCCCTgTgAgTCCTTCAg-3′	NM_009386.2
*(331 bp, 55°C, A:10s, E:15s)*	Rev: 5′-CCAggTTTTAgggTCACAgT-3′	
	Red: R610-ATgCCACgAgCTgTAgCCACTAC-Phos	
	FL: 5′-CTCAACACACCACCATTgCTgTT-FL	
ZO-1 isoform 2 (ZO-1 V2)	Forw: 5′-TgTCCCTgTgAgTCCTTCAg-3′	NM_001163574.1
*(330 bp, 55°C, A:10s, E:15s)*	Rev: 5′-ggAgTCATggACgCACAgT-3′	
	Red: R610-ATgCCACgAgCTgTAgCCACTAC-Phos	
	FL: 5′-CTCAACACACCACCATTgCTgTT-FL	
Cyclophilin A (PPIA)	Forw: 5′-gCgTCTSCTTCgAgCTgTT-3′	NM_008907
*(146 bp, 55°C, A:10s, E:15s)*	Rev: 5′-RAAgTCACCACCCTggCA-3′	[15]
	Red: R610-TTggCTATAAgggTTCCTCCTTTCACAg-Phos	
	FL: 5′-gCTCTgAgCACTggRgAgAAAggA-FL	

Forw = sense primer; Rev = anti-sense primer, R610 = Lightcycler® Red610, Phos = phosphate, FL = fluorescein.

### Immunohistochemistry

24 hours after CCI or sham surgery the initial anesthetic regimen was repeated for deep anesthesia and the animals were transcardially perfused using 4% paraformaldehyd (PFA). The brains were carefully removed and postfixed in 4% PFA for 24 hours. Subsequently brains were washed in 0.01 mol/l PBS and incubated in sucrose (30% in 0.01 mol/l PBS) overnight at RT under stiring. Brains were cut into 100 µm thick slices with a freezing microtome (Leitz CM 1325, New York, NY, USA). For co-stainings of ZO-1, cl5 and counterstaining of cell nuclei with 4′,6-Diamidino-2-phenylindole dihydrochloride (DAPI from Sigma, 1∶2000) mice were transcardially perfused with PBS. Then brains were removed, embedded in tissue tek and snap frozen. Frozen brains were cryosectioned (10–20 µm) and fixed in ice-cold acetone. Slices were blocked and permeabilized with 7% normal goat serum (Jackson ImmunoResearch via Dianova, Hamburg, Germany) and 0.3% triton (Sigma-Aldrich Steinheim, Germany) in PBS 0.01 mol/l for 2 hours at RT. For immunohistochemical staining slices were incubated with the primary antibodies (1∶100 rabbit anti-ZO-1 or 1∶50 mouse anti-claudin-5, Invitrogen via Zymed) in 2% bovine serum albumin (Dianova) with 0.05% azide and 0.1% Triton in PBS 0.01 mol/l overnight at RT. Then probes were incubated with secondary antibodies (CyTM2-conjugated AffiniPure goat anti-rabbit IgG H+L, Dianova; or Alexa Fluor 568 goat anti-mouse, molecular probes) diluted in 2% bovine serum albumin for 2 hours at RT. Probes were washed in PBS 0.01 mol/l and embedded in Fluoromount (Southern Biotech, Birmingham, AL, USA) or Fluorsave (Calbiochem San Diego, CA, USA).

For cl5 staining in PFA fixed probes sections were unmasked with citric acid monohydrate (Roth, Karlsruhe, Germany) 0.01 mol/l pH 6.0 on superfrost-slides (Menzel, Braunschweig, Germany) in the microwave (boiling ∼2 minutes) ahead of antibody application.

### Immunofluorescence confocal microscopy (ICM)

An upright microscope (BX51WI, Olympus; Hamburg, Germany) with a Nipkow spinning disk confocal system (QLC10 Visitech, Sunderland, United Kingdom) and a Krypton/Argon laser (Laser Physics, Cheshire, UK) was used for ICM. Fluorescent probes were excited at 488 nm and 568 nm wavelength. For three-color analyses of cl5, ZO-1, and DAPI a Leica SP5 confocal laser-scanning microscope was used. Here excitations were 405 nm, 488 nm, and 561 nm wavelength. Images were analyzed and processed with Leica Application Suite Advanced Fluorescence (Leica Microsystems), Metamorph (Molecular Devices Corp., Downington, CA, USA) and ImageJ (NIH software).

### Quantification of TJ gap formation in CCI

ZO-1 and cl5 gap formation was analyzed as described before [11] with modifications using ImageJ software. The length of ZO-1 or cl5 disruptions was related to the total length of cerebral microvessels and TJ gap formation was calculated as percentage of the total number of investigated TJs. For statistical analyses 20–26 microvessels from 3 brains were analyzed per group in the pericontusional area.

### Statistical Analysis

Statistical analysis was performed with Sigma Plot 11 statistical software (SPSS Science, Chicago, USA) and Graph Pad Prism (Windows version 4.02, GraphPad Software, San Diego, CA, USA). TEER and microvascular measurements were analyzed by ANOVA and post hoc Tukey tests (study A and D). Wilcoxon Mann Whitney Rank Sum Tests were used in study B, C–E and p-values were adjusted for multiple comparisons (Bonferroni adjustment). A value of P<0.05 was considered as significant. Results are presented as mean ± standard deviation (SD).

## Results

### Effect of anesthetics on in-vitro blood brain barrier integrity

**Figure 1:**
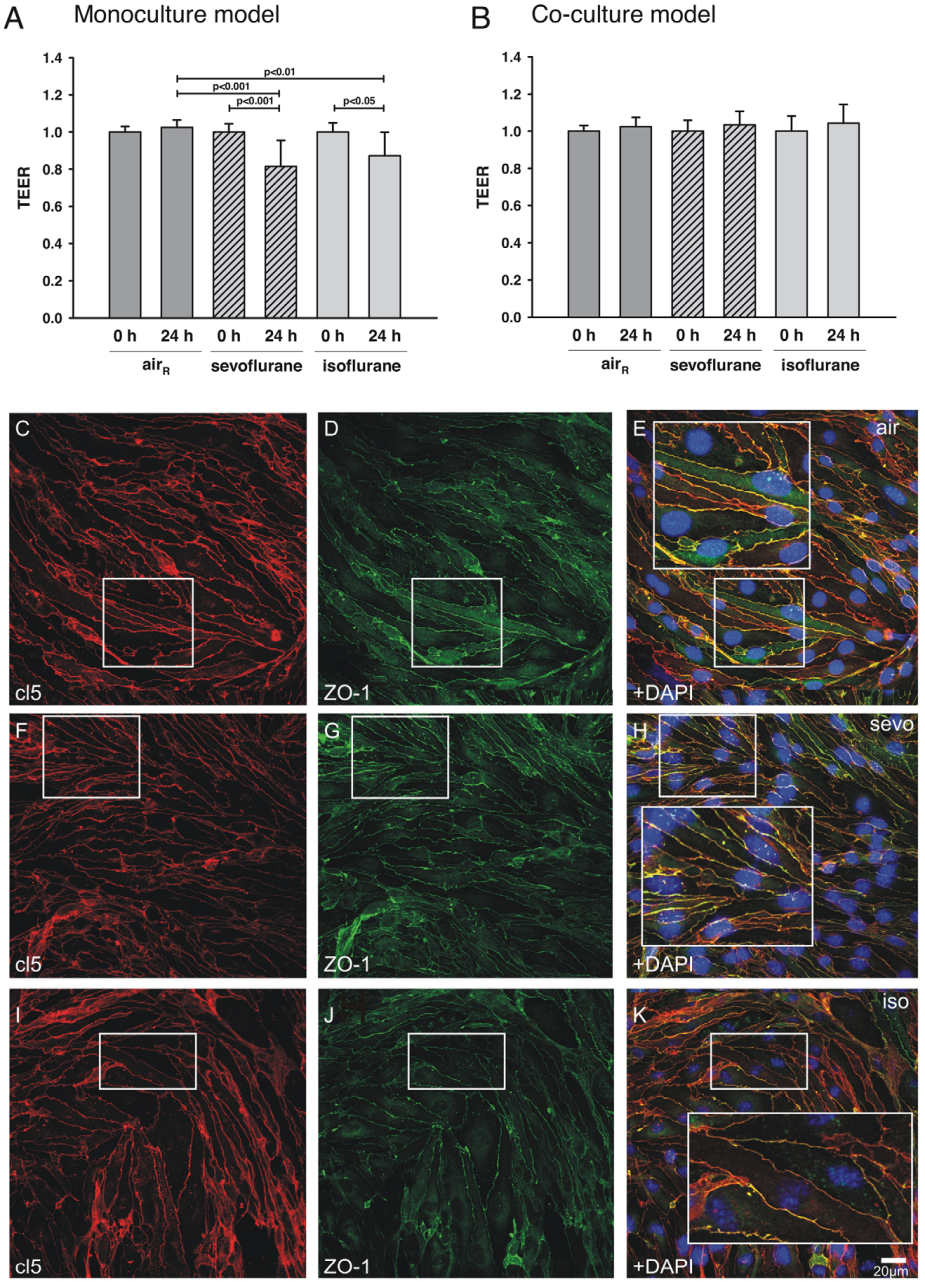
Effect of isoflurane and sevoflurane on TEER in vitro. (**A**) In a monoculture model bEnd.3 cells were exposed for 20 minutes to room air only (air_R_), air_R_ containing 1.5% of either isoflurane or 3.5% of sevoflurane. After 24 hours TEER values were evaluated. air_R_ had no effect on TEER whilst iso and sevo significantly reduced TEER values. (**B**) In an in vitro co-culture model of the BBB that includes most cell types of the NVU isoflurane or sevoflurane had no significant impact on TEER. Immunohistochemical analyses confirmed that BBB integrity within the BBB co-culture model was not affected by volatile anesthetics. (**C–E**) Probes exposed to air only demonstrated a continuous alignment and co-localization of cl5 and ZO-1 in the BBB co-culture model. No differences in cellular integrity of cl5 and ZO-1 were found in probes treated with (**F–H**) sevoflurane or (**I–**K) isoflurane. (**E, H, K**) Note the physiological co-localization of cl5 and ZO-1.

To analyze BBB integrity TEER values at 0 hours before gas exposure and 24 hours after gas exposure TEER values were evaluated, and for normalization purposes the values for both time points were set in relation to average TEER values before gas exposure, respectively (**Figure 1**). In a monoculture model both volatile anesthetics isoflurane (0 h = 1±0.05 vs. 24 h = 0.87±0.13, P<0.05, n = 8) and sevoflurane (0 h = 1±0.04 vs. 24 h = 0.82±0.14, P<0.001, n = 8) significantly reduced TEER (**Figure 1 A**) after 24 hours of gas exposure (air_R_ 24 h = 1.03±0.04 vs. isoflurane 24 h 0.87±0.13, P<0.01; air_R_ 24 h = 1.03±0.04 vs. sevoflurane 24 h = 0.82±0.14, P<0.001, n = 8). Although the reduction of TEER was higher after sevo exposure, there were no significant differences between the two volatile anesthetics. Room air had no significant effect on TEER values after 24 h (air_R_ 0 h = 1±0.03 vs. air_R_ 24 h = 1.03±0.04, P>0.05, n = 8).

In the co-culture model (**Figure 1 B**) volatile anesthetics and room air had no significant impact on TEER (isoflurane 0 h = 1±0.08 vs 24 h 1.04±0.1; sevoflurane 0 h = 1±0.06 vs. 24 h = 1.04±0.07; room air 0 h = 1±0.03 vs. room air 24 h 1.02±0.05, P>0.05 in all groups, n = 8) and ZO-1 or cl5 integrity (Figure 1 C–K).

### Effect of anesthesia on brain water content and TJ protein expression in native animals

**Figure 2:**
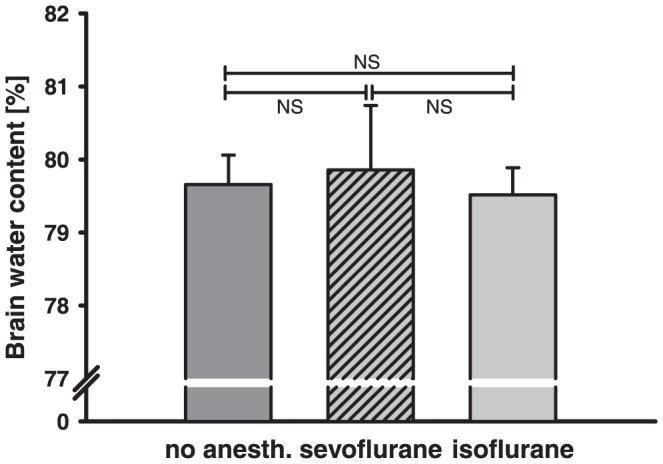
Brain edema formation in native animals. 24 hours after 20 minutes exposure with 1.5% isoflurane, 3.5% sevoflurane or pure O_2_/N_2_ mixture (no anesth.) exposure brain water content was determined in native animals. The anesthesia exposure did not influence brain water content.

**Figure 3:**
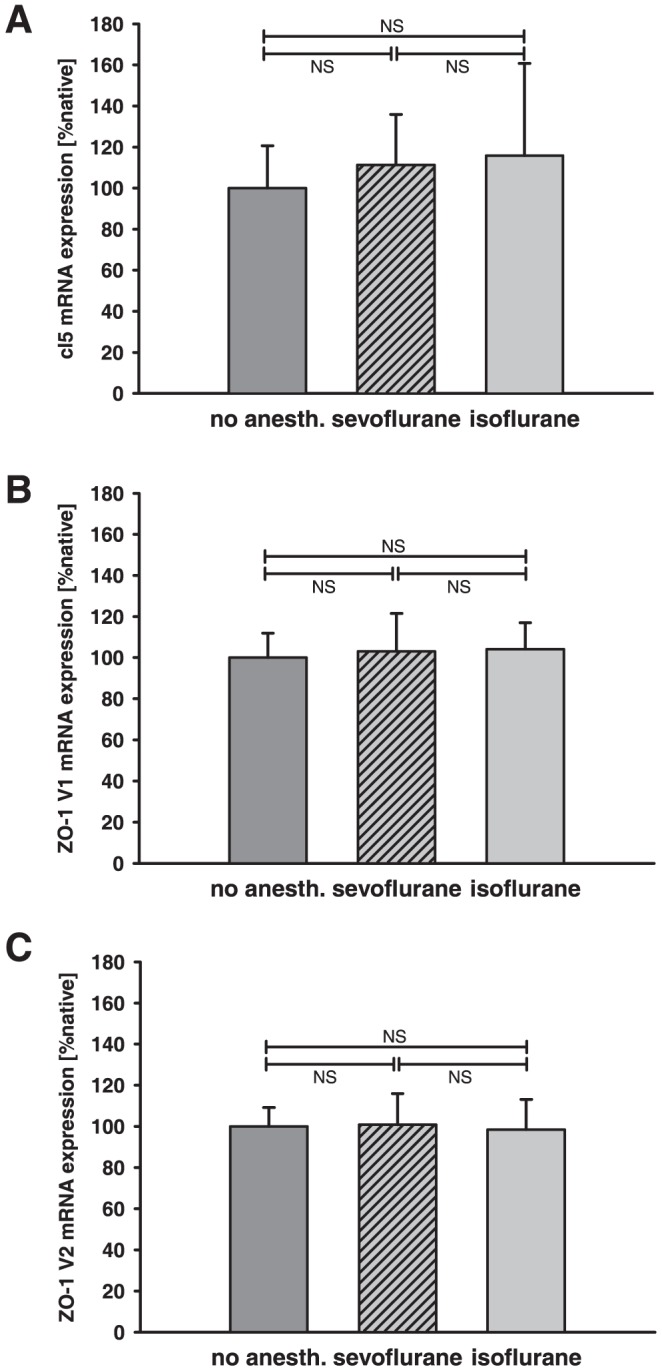
Influence of isoflurane and sevoflurane on TJ protein expression in native animals. Expression of the TJ proteins cl5 (A), ZO-1 isoform 1 (ZO-1 V1, B), and ZO-1 isoform 2 (ZO-1 V2, C) was determined 24 hours after exposure to isoflurane and sevoflurane and compared to no anesthesia (same O_2_/N_2_ mixture). The expression of the TJ proteins was not significantly different between groups (n = 8/group; p-values are adjusted for multiple comparison by Bonferroni).

To investigate the impact of anesthesia on brain water content in healthy animals, hemispherical water content was determined by measuring the wet/dry ratio (**Figure 2**). 1.5% isoflurane, 3.5% sevoflurane or no anesthesia was initiated for 20 minutes. 24 hours after anesthesia exposure brain water content was not significantly different between the experimental groups (no anesthesia [40% O_2_] = 79.66±0.4%, isoflurane = 79.51±0.4%, sevoflurane = 79.86±0.9%). Expression of the TJ proteins cl5, ZO-1 isoform 1 (ZO-1 V1), and ZO-1 isoform 2 (ZO-1 V2) was not significantly different in native animals 24 hours after anesthesia exposure (**Figure 3**).

### Effect of anesthesia on brain water content after CCI

**Figure 4:**
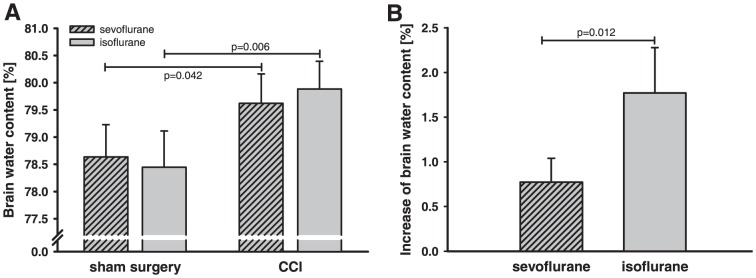
Brain edema formation in traumatized mice. (**A**) Brain water content was determined 24 hours after CCI or sham operation. The water content of the right hemisphere in the sevoflurane and isoflurane group increased significantly in comparison to sham operated animals. Increase of brain water content differed depending on anesthetics. (**B**) For the absolute increase of brain water content, the difference of brain water content of sham operated and traumatized animals exposed to the same anesthetics was calculated. Compared to sham values, increase in brain water content was significantly higher with isoflurane compared to sevoflurane.

In order to investigate if anesthetics influence brain water content after an acute cerebral lesion wet/dry ratio was determined 24 hours after CCI or sham surgery (**Figure 4**). The water content of the injured hemisphere increased significantly in both groups (sevoflurane+CCI = 79.62±0.5%, P<0.05; isoflurane+CCI = 79.89±0.5%, P<0.01) 24 hours after CCI in comparison to sham-operated animals (sevoflurane+sham surgery = 78.64±0.6%; isoflurane+sham surgery = 78.45±0.7%). Compared to sham operated animals, brain water content of traumatized animals demonstrated a significant higher increase with isoflurane (+1.77±0.5%, P<0.05) compared to sevoflurane (+0.77±0.3%).

### Tight junction integrity in the pericontusional region

**Figure 5:**
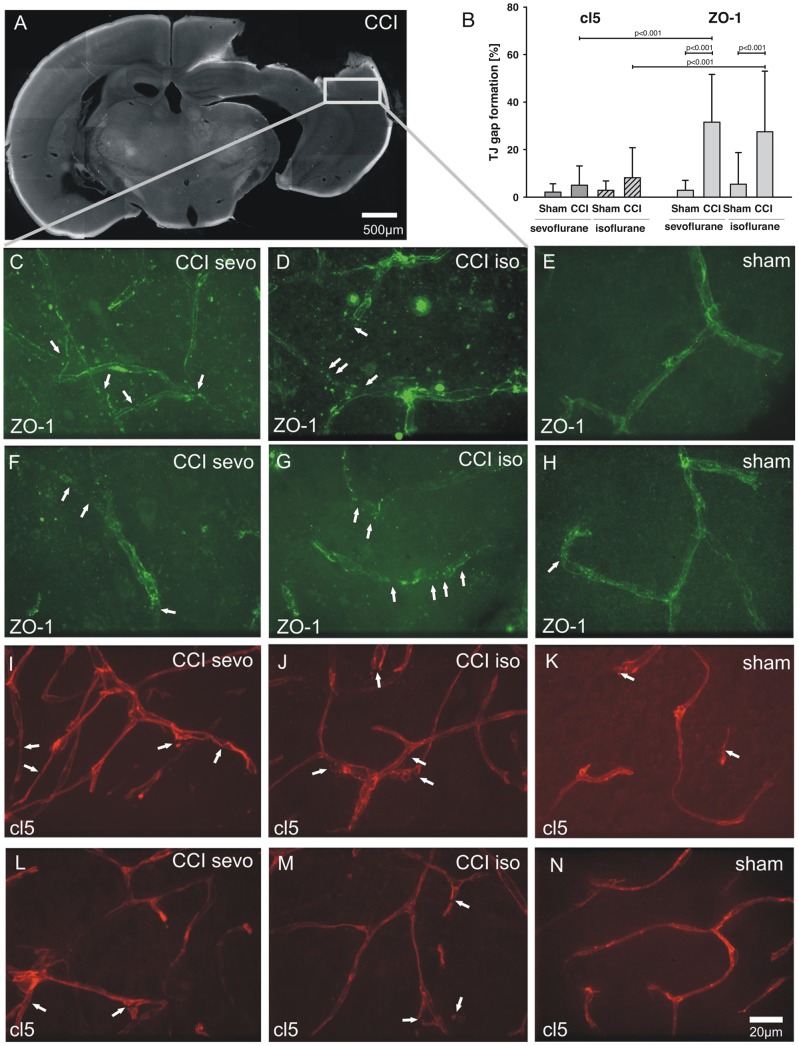
Immunohistochemical analysis of ZO-1 and cl5 gap formation in the pericontusional brain area. (**A**) Immunohistochemical analyses were performed in the pericontusional cortical area (marked by the grey box) of CCI and (**B**) sham brains. (**B**) CCI resulted in heavy ZO-1 impairment in cerebral microvessels while cl5 was not significantly impaired (ZO-1 sevoflurane CCI 31.6±20.1% vs. ZO-1 sevoflurane sham 2.9±4.2%, P<0.001, n = 20 microvessels, 3 brains per group; ZO-1 isoflurane CCI 27.5±25.5% vs. ZO-1 isoflurane sham 5.5±13.3%, P<0.001, n = 22 microvessels, 3 brains per group; cl5 sevoflurane CCI 5±8.1% vs. cl5 sevoflurane sham 2.1±3.5%, P>0.05, n = 21–26 microvessels, 3 brains per group; cl5 isoflurane CCI 8.2±12.7% vs. cl5 isoflurane sham 2.9±3.9%, P>0.05, n = 21–22 microvessels, 3 brains per group). Impairment of ZO-1 in CCI was significantly higher than cl5 damage in sevoflurane (ZO-1 sevoflurane CCI 31.6±20.1% vs. cl5 sevoflurane CCI 5±8.1%, P<0.001, n = 20–26 microvessels, 3 brains per group) and isoflurane (ZO-1 isoflurane CCI 27.5±25.5% vs. cl5 isoflurane CCI 8.2±12.7%, P<0.001, n = 22 microvessels, 3 brains per group) groups. ZO-1 and cl5 gap formation was not significantly affected during CCI by the choice of the volatile anesthetic (ZO-1 sevoflurane CCI 31.6±20.1% vs. ZO-1 isoflurane CCI 27.5±25.5%, P>0.05, n = 20–22 microvessels, 3 brains per group) or iso (cl5 sevoflurane CCI 5±8.1% vs. cl5 isoflurane CCI 8.2±12.7%, P>0.05, n = 22–26 microvessels, 3 brains per group). (**C–N**) Representative confocal images of TJ damage. Numerous ZO-1 disruptions were found in CCI animals treated with sevoflurane (panels **C** and **F**) or isoflurane (**D** and **G**). ZO-1 discontinuities in sham animals were sparse (representative images of ZO-1 in sham animals in **E** and **H**). Only few cl5 disruptions were present in CCI in both sevoflurane (**I** and **L**) and isoflurane (**J**, **M**) in comparison to sham brains (representative images of cl5 in sham animals in **K**, **N**).

**Figure 6:**
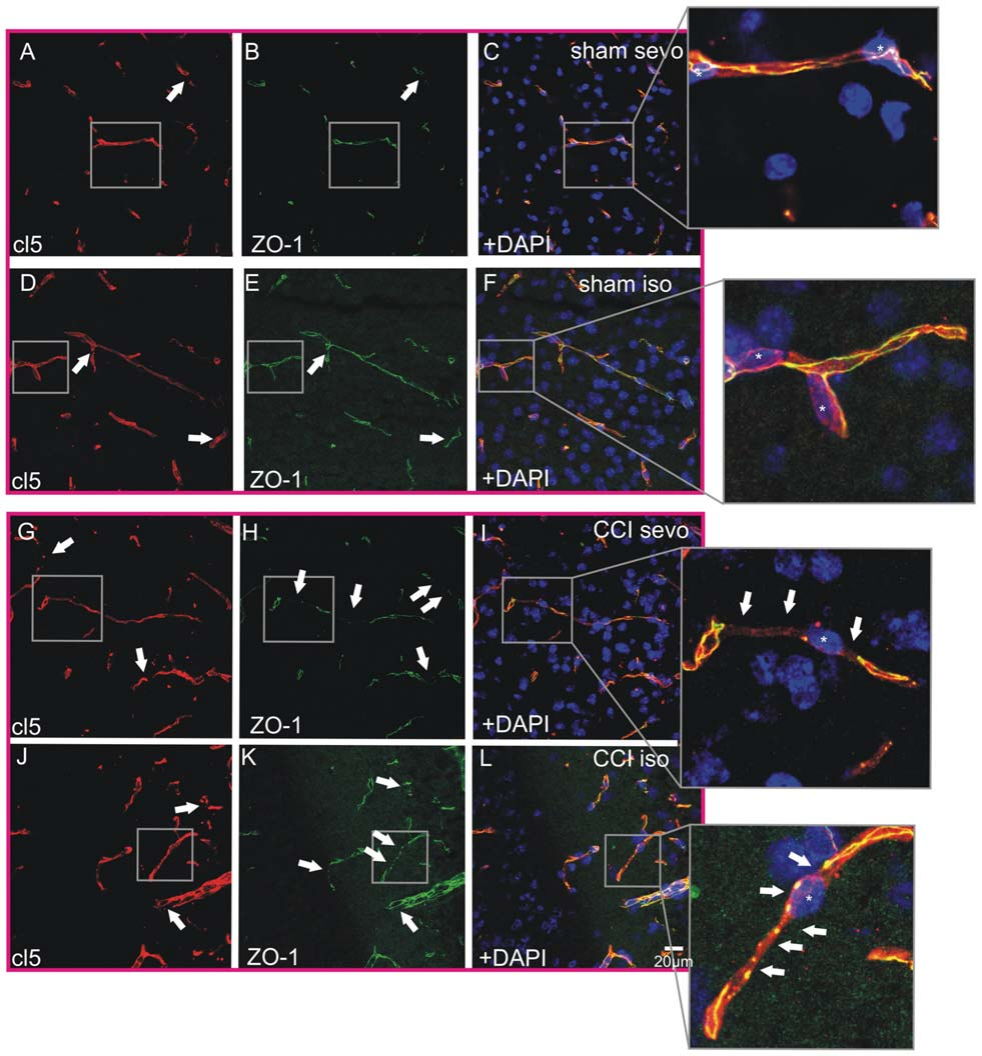
Triple-staining of DAPI, ZO-1 and cl5 in the pericontusional brain area of CCI and sham animals. (**A**) cl5 and (**B**) ZO-1 immunoreactivity in sham sevoflurane treated animals demonstrate a physiological co-localization as shown in (**C**). In sham animals exposed towards isoflurane (**D**) cl5 and (**E**) ZO-1 displayed a similar physiological (**F**) co-localization of the junctional proteins. TJ disruptions in sham animals were sparse as indicated by the white arrows in A–F. In CCI brains of the sevoflurane group a significant discrepancy of (**G**) cl5 and (**H**) ZO-1 became apparent as shown in (**I**). In the isoflurane CCI group (**J**) cl5 was also less affected by CCI than (**K**) ZO-1. Here a similar discrepancy as in CCI sevoflurane treated animals was found (**L**). TJ disruptions in ZO-1 were significantly increased compared with ZO-1 sham animals. No difference in cl5 disruptions was observed (G–L, see also quantification of TJ disruptions in Figure 5). Note the microvascular brain endothelial nuclei marked with asterisks (insets in **C, F, I, L**).

Tight junction protein integrity is a hallmark for brain edema progression in pathologic conditions such as stroke [16]. It has been documented that the TJ proteins ZO-1 and cl5 have a pivotal role for the integrity of the BBB in this context [17]. To elucidate whether these proteins also have a major role in CCI, the pericontusional area of CCI brains (**Figure 5 A**) was analyzed quantitatively for ZO-1 and cl5 gap formation in cerebral microvessels (**Figure 5 B**). ZO-1 was more affected by CCI than cl5 in both sevoflurane (ZO-1 sevoflurane CCI 31.6±20.1% vs. cl5 sevoflurane CCI 5±8.1%, P<0.001, n = 20–26 microvessels, 3 brains per group) and isoflurane (ZO-1 isoflurane CCI 27.5±25.5% vs. cl5 isoflurane CCI 8.2±12.7%, P<0.001, n = 22 microvessels, 3 brains per group). The choice of the volatile anesthetics isoflurane or sevoflurane did not significantly alter cl5 or ZO-1 gap formation after CCI (ZO-1 sevoflurane CCI 31.6±20.1% vs. ZO-1 isoflurane CCI 27.5±25.5%, P>0.05, n = 20–22 microvessels, 3 brains per group; cl5 sevoflurane CCI 5±8.1% vs. cl5 isoflurane CCI 8.2±12.7%, P>0.05, n = 22–26 microvessels, 3 brains per group). Further, we compared ZO-1 and cl5 TJ impairment in sham animals with the CCI groups. Here it became apparent that ZO-1 is significantly disrupted in the pericontusional area whilst cl5 is not significantly altered in the pericontusional zone compared with sham animals in isoflurane as well as in sevoflurane groups (ZO-1 sevoflurane CCI 31.6±20.1% vs. ZO-1 sevoflurane sham 2.9±4.2%, P<0.001, n = 20 microvessels, 3 brains per group; ZO-1 isoflurane CCI 27.5±25.5% vs. ZO-1 isoflurane sham 5.5±13.3%, P<0.001, n = 22 microvessels, 3 brains per group; cl5 sevoflurane CCI 5±8.1% vs. cl5 sevoflurane sham 2.1±3.5%, P>0.05, n = 21–26 microvessels, 3 brains per group; cl5 isoflurane CCI 8.2±12.7% vs. cl5 isoflurane sham 2.9±3.9%, P>0.05, n = 21–22 microvessels, 3 brains per group). No significant differences in TJ damage were observed in sham groups treated with sevoflurane or isoflurane. The pericontusional area of CCI and sham brains (**Figure 5**) was analyzed with ICM. Prominent ZO-1 disruptions were detected in the pericontusional area in animals that were treated with sevoflurane (**Figure 5 C** and **F**) or isoflurane (**Figure 5 D** and **G**) during CCI induction. In sham isoflurane or sevoflurane animals only slight ZO-1 discontinuities were found (**Figure 5 E** and **H**). Fewer cl5 disruptions were present in CCI brains that were exposed to sevoflurane (**Figure 5 I** and **L**) or isoflurane (**Figure 5 J** and **M**). Only sparse cl5 dislocation was present in sham animals (**Figure 5 K** and **N**). Apparently, ZO-1 was more affected by CCI in the pericontusional zone than cl5. To better evaluate the discrepancy observed in cl5 and ZO-1 impairment we performed triple labeling of cl5, ZO-1 and cell nuclei with DAPI. Representative co-stainings of 3 animals per group are shown. In sham groups treated with sevoflurane (**Figure 6 A–C**) or isoflurane (**Figure 6 D–F**) a continuous TJ expression in cortical microvessels with only sparse TJ disruptions (arrowheads in **A–E**) were present. In both sham groups cl5 and ZO-1 co-localized in a physiological manner (**C** and **F**). In CCI groups treated with sevoflurane cl5 (**G**, arrowheads) was less disrupted than ZO-1 (**H**, arrowheads) as demonstrated in the merged image in **I**. A similar result was obtained in CCI animals treated with isoflurane. Here, cl5 integrity (**J**, arrowheads) was also much less affected than ZO-1 (**K**, arrowheads) as shown in the overlay in **L**. However, both junctional proteins cl5 and ZO-1 displayed disruptions within the CCI core region (data not shown).

### Tight junction mRNA expression in the pericontusional region

**Figure 7:**
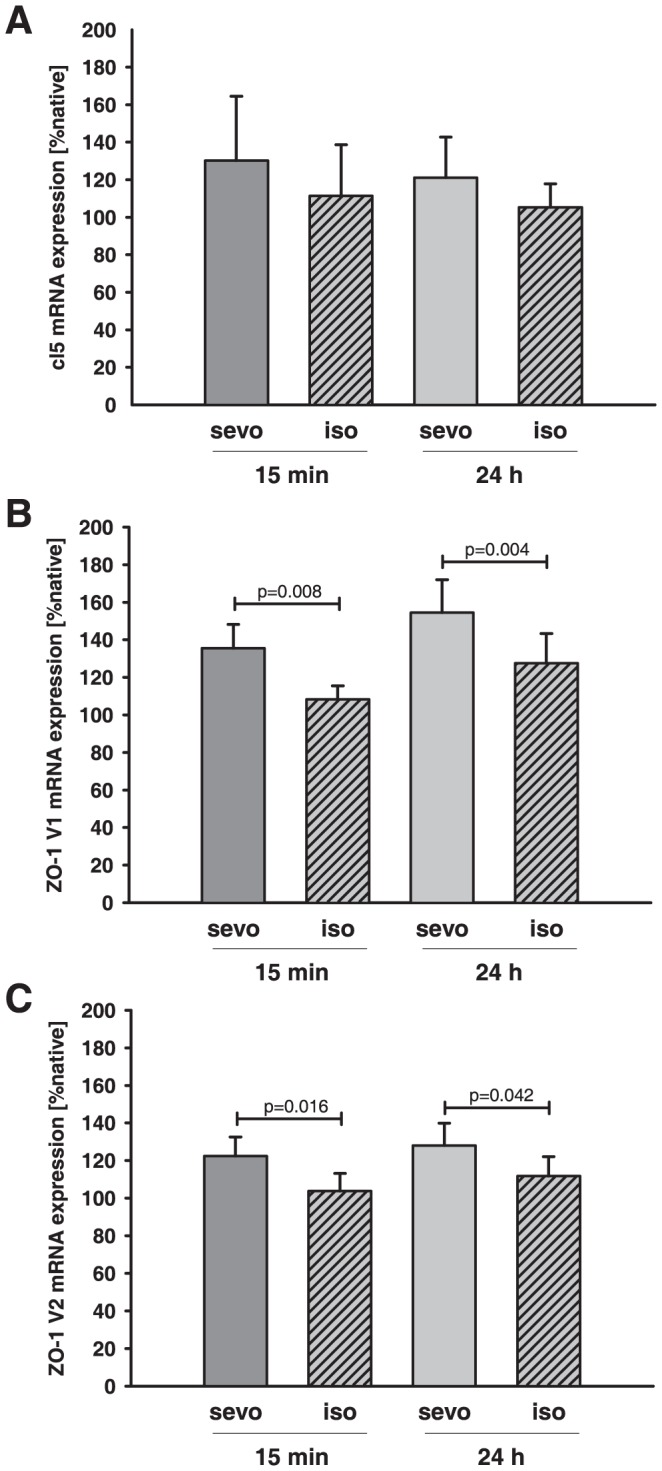
Influence of isoflurane and sevoflurane on TJ protein expression after CCI. Expression of the TJ proteins cl5 (A), ZO-1 isoform 1 (ZO-1 V1, B), and ZO-1 isoform 2 (ZO-1 V2, C) was determined at 15 minutes and 24 hours after CCI injury and exposure to isoflurane (iso)/sevoflurane (sevo) and is expressed as % of native animals (n = 6/15 min groups; n = 8/24 h groups; p-values are adjusted for multiple comparison by Bonferroni).

In contused brain tissue mRNA content of cl5 was not significantly altered 15 minutes and 24 hours after trauma both for the sevoflurane as well as for the isoflurane group (**Figure 7** **A**). The amount of ZO-1 isoform 1 (ZO-1 V1: 15 min sevoflurane = 131±15% native vs. isoflurane = 108±7% native; 24 h sevoflurane = 154±18% native vs. isoflurane = 128±16% native, **Figure 7** **B**) and ZO-1 isoform 2 (ZO-1 V2: 15 min sevoflurane = 122±10% native vs. isoflurane = 104±9% native; 24 h sevoflurane = 123±18% native vs. isoflurane = 112±10% native, **Figure 7** **C**) copies was significantly higher in the sevoflurane group compared to the isoflurane group at all time points.

## Discussion

Blood-brain barrier breakdown results in brain edema formation, which is one major cause for the high mortality rate after severe brain injury. Tight junction proteins such as ZO-1 and cl5 play a critical role to maintain BBB function by improving the barrier function at the endothelial level [10], [11], [18]. Our data suggest that anesthetics differentially influence BBB permeability. The key findings of this study are: 1.) Sevoflurane and isoflurane reduce transendothelial electrical resistance of in-vitro BEC but not in a more complex co-culture model of the BBB; 2.) Following TBI brain water content in isoflurane-anesthetized animals was higher than in brains of sevoflurane-treated animals; 3.) ZO-1 integrity on the cellular level is more affected by TBI than cl5 whilst isoflurane or sevoflurane had no impact on microvascular TJ integrity; 4.) Sevoflurane application is associated with a stronger ZO-1 expression after TBI compared to isoflurane. The data therefore indicate that volatile anesthetics differentially influence the integrity of BBB for a prolonged phase after exposure.

An intact BBB is essential for the fluid and substrate homeostasis of the brain and disruption results in disturbance in regulation of brain water balance and consecutive brain edema formation. Brain edema is considered to be one of the central pathophysiological events worsening outcome by raising intracranial pressure and impairing cerebral perfusion [2].

Following TBI anesthetics are routinely applied during intensive care or surgical procedures; and they are even utilized to reduce cerebral metabolisms and blood volume during phases of elevated ICP. So far, it is unclear if volatile anesthetics are capable of inducing BBB impairment at the endothelial level. However it has been recently demonstrated that in an in vitro model of astrocyte-conditioned human umbilical vein endothelial cells isoflurane is capable of inducing apoptosis [19]. The bEnd.3 cell line is a model of murine cerebrovascular endothelium maintaining several in-vivo properties of the BBB like expression of tight junction proteins [10], [20], permeability to sucrose, high trans endothelial electrical resistance as well as transport of glucose [20]. The neurovascular in vitro co-culture consists of most cell types of the neurovascular unit (NVU) including neurons and astrocytes. In the bEnd.3 monoculture model, exposure towards the volatile anesthetics isoflurane and sevoflurane induced a decrease of TEER. TEER correlates with BBB integrity in vitro [19]. At this point we would like to stress the fact that absolute TEER values evaluated in ECIS are not comparable to TEER values evaluated in cell culture inserts that include porous membranes. A detailed description of the method itself can be found in the work by Tirppathi et al. [21]. However, we have previously demonstrated that impaired TEER values in ECIS within bEnd.3 monolayers as well as the neurovascular BBB model go along with disruptions in cl5 and ZO-1 [10], [11].

Volatile anesthetics like isoflurane and sevoflurane are potent direct vasodilators [22] – vasodilatation of the vessels, induced by isoflurane has been reported to initiate BBB disruption [7]. In contrast to the in-vitro monoculture results, the brain water content in sham operated and in native animals did not differ significantly independent of exposure to volatile anesthetics. Further we did not observe a significant impact of sevoflurane or isoflurane on TEER and TJ integrity within an in-vitro model that consists of most cell types of the neurovascular unit [10]. This is in agreement with our observation that BBB function does not seem to be affected in native animals exposed to sevoflurane or isoflurane. Healthy brain tissue and a complex model of the BBB therefore seem to compensate changes introduced by the application of both volatile anesthetics, while in-vitro brain endothelial cells are not able to solely maintain barrier function upon volatile anesthetic stimulation. The in-vitro model of a monoculture of bEnd.3 is therefore a very sensitive tool to detect BBB dysfunction, but in the experimental setting of the present study it overestimates the potential effect in healthy brain tissue. Most likely the reason is that it does not represent a functional neurovascular unit as it is found in vivo [10]. However, TBI results in spreading depolarization that significantly perturb the physiologic interplay of the NVU [20], which may also make the brain endothelium more vulnerable towards injury. Our data suggest that in conditions where some cell types of the NVU are lacking or are not physiologically regulated, an enhanced level of vulnerability towards the brain endothelium is present.

Volatile anesthetics have been shown to influence edema formation in injury models in other organ systems, e.g. a recent study demonstrated that the development of lung edema was enhanced by isoflurane and not by sevoflurane in an animal model of pulmonary injury [23]. Isoflurane in conjunction with mechanical ventilation blunted cardiovascular compensatory mechanisms in sepsis and enhanced leukocyte activation, which may also contribute to lung edema formation [24]. Possibly, in the context of TBI anesthesia may also have an impact on brain water content following experimental head injury. The time-course experiments of brain edema formation following brain trauma demonstrate an increase of brain water content already 2 hours after insult with a maximum after 24 hours [25], [26]. The disruption of the BBB integrity occurs immediately after insult and reaches a maximum after 4–6 hours and a second peak 3 days after brain injury [25]. In the present study brain water content increased significantly after trauma in all groups compared to sham operated animals 24 hours after brain trauma. The extent of increase differed depending on the anesthetics - being highest with isoflurane and lowest with sevoflurane. Apart from neuroprotective effects of volatile anesthetics [27], [28], different anesthetics also increase cerebral water content in animals with or without brain injury [7]. Currently no data is available on the effect of sevoflurane on BBB integrity and brain edema formation during TBI. Animal studies indicate that isoflurane anesthesia impairs BBB [7], increases plasma glutamate levels and brain water content in brain-injured rats [29] and raises plasma glutamate levels in neurosurgical patients [30]. Isoflurane induces a larger brain edema formation in comparison to pentobarbital in experimental cerebral ischemia [31]. The potential edema enhancing effect of isoflurane is dependent upon concentration, time point, and duration of exposure [7]. An increase in bi-hemispherical water content was observed in traumatized rats subjected to 4 hours of isoflurane anesthesia [29]. Contrary to this finding a low concentration of isoflurane did not result in an increase in posttraumatic brain edema in rats [32]. In non-traumatized dogs anesthetics also have been shown to increase cerebral water content [33]. Already a short duration of isoflurane increased water content in the pyriform cortex in uninjured rat brains [34]. Multiple data therefore demonstrate a modulatory effect of anesthetics on BBB function – which may be caused by their direct action on cerebral vessels. An increase of cerebral blood flow (CBF) and vasodilation is associated with an increase of brain water content [35]. Volatile anesthetics have effects on cerebral vasodilation, cerebral blood volume, CBF, and ICP in a dose dependent manner [36], [37]. During propofol-induced isoelectric electroencephalography isoflurane treatment in patients resulted in a higher CBF in the middle cerebral artery than under sevoflurane anesthesia [36]. Sevoflurane has been reported to decrease CBF in a dose dependent manner in pigs [38] in contrast to data on isoflurane reported by the same group [39]. On the contrary sevoflurane and isoflurane have been shown to have similar effects on CBF in animals and humans [22], [40], [41]. Therefore, the vasodilatory effects of these anesthetics cannot explain the observed differences in this study.

The permeability of the BBB is modulated by the cellular localization, expression and by protein-protein interaction of the TJ proteins. A wide array of growth factors, cytokines, drugs, caspase-3 [11], matrix metalloproteinases [42], and hormones influence tight junctions and barrier function. For example, steroids [43] or unsaturated fatty acids [44] enhance the tight junction tightness by increasing the expression of occludin. Histamin, caspase-3 [11], cytokines, VEGF (review: [45]), and tumor necrosis factor-α [46] perturb TJ integrity by decreasing occludin and ZO-1 expression and causing cl5 and ZO-1 protein disruption. TJs are regulated by a diverse group of extracellular stimuli that initiate various intracellular signaling cascades. Further, ischemic [47] or traumatic [48] insults reduced both ZO-1 and occludin levels or resulted in impaired TJ alignment [11]. In the present study TJ proteins critical to maintain BBB function (cl5 and ZO-1) were investigated. TBI resulted in a disruption of ZO-1 and cl5 protein interaction 24 hours following CCI. ZO-1 was more susceptible towards cellular disturbances after TBI than cl5. The discrepancy between ZO-1 and cl5 impairment is an important finding: recently it has been documented that a targeted suppression of cl5 is beneficial for outcome and reduces brain edema evolution in vivo [18]. These results suggest that the persistence of cl5 in the pericontusional zone may be of pivotal importance for outcome in TBI. Isoflurane and sevoflurane did not have an effect on these changes. However, the mRNA expression of ZO-1 was higher in the sevoflurane group compared to native and isoflurane treated animals. Isoflurane did not significantly alter TJ protein cl5 and ZO-1 expression on the mRNA level.

The present data demonstrate that isoflurane treatment resulted in a significant elevation in brain water content in a model of TBI. In contrast lower brain water levels were found after treatment with sevoflurane. Sevoflurane increased ZO-1 mRNA expression which was beneficial for BBB integrity as reflected by a reduced brain edema in sevoflurane treated animals after TBI. The present data suggest that the difference in extent of brain edema formation after brain trauma by use of sevoflurane for anesthesia may be dependent upon ZO-1 impairment on the cellular and mRNA level. Although TJ proteins, especially ZO-1, are disrupted after TBI, a higher mRNA expression of ZO-1 correlated with less increase in brain water content.

## Conclusion

The study demonstrates that isoflurane and sevoflurane influence brain edema formation after experimental brain injury and the expression of ZO-1 on mRNA level. Therefore, the selection of anesthetics may influence the barrier function. The observed effect indicate 1.) a strong bias introduced by the selection of the anesthesia to experimental research on pathophysiological mechanisms of BBB dysfunction and 2.) a possible clinical implication to modulate the BBB function by selection of sevoflurane as anesthetic agent during posttraumatic surgical procedures.
